# Social transmission of tool use and tool manufacture in Goffin cockatoos (*Cacatua goffini*)

**DOI:** 10.1098/rspb.2014.0972

**Published:** 2014-10-22

**Authors:** A. M. I. Auersperg, A. M. I. von Bayern, S. Weber, A. Szabadvari, T. Bugnyar, A. Kacelnik

**Affiliations:** 1 Department of Cognitive Biology, University of Vienna, Althanstrasse 14, Vienna 1190, Austria; 2 Department of Zoology, University of Oxford, Oxford OX1 3PS, UK; 3 Max Planck Institute for Ornithology, Eberhard-Gwinner-Strasse 4, Seewiesen 82319, Germany

**Keywords:** social learning, tool use, tool manufacture, emulation, imitation, avian cognition

## Abstract

Tool use can be inherited, or acquired as an individual innovation or by social transmission. Having previously reported individual innovative tool use and manufacture by a Goffin cockatoo, we used the innovator (Figaro, a male) as a demonstrator to investigate social transmission. Twelve Goffins saw either demonstrations by Figaro, or ‘ghost’ controls where tools and/or food were manipulated using magnets. Subjects observing demonstrations showed greater tool-related performance than ghost controls, with all three males in this group (but not the three females) acquiring tool-using competence. Two of these three males further acquired tool-manufacturing competence. As the actions of successful observers differed from those of the demonstrator, result emulation rather than high-fidelity imitation is the most plausible transmission mechanism.

## 1. Introduction

Tool-related behaviour is not only phylogenetically widespread but also rare and hard to implement in artificial systems. Because of this, and because of the long tradition of seeing tool use and tool making as quintessentially human traits, the use and manufacture of tools are often assumed to be associated with sophisticated cognitive traits. Today, we know that this generalization is not valid, as tool use sometimes involves stereotyped, more or less inherited motor patterns which are not adaptable to novel circumstances, and hence do not seem to demand complex cognitive processing (e.g. [1,2]). Nevertheless, in some cases, particularly in birds and mammals, tool use involves flexible behaviours, which are not represented in the species' heritable inventory of adaptations to ecologically frequent problems (e.g. [1,2]).

A further important distinction is between-individual innovations, when subjects use objects in a novel way to overcome unusual problems, and socially acquired tool use, where tool-related skills are acquired through observation of and/or interactions with others. The latter can be revealing of the cognitive underpinnings of social learning mechanisms and the processing of information about self and others, which are core issues for research on comparative cognition and the evolution of culture. Social learning can reveal the extent to which an observer (i) is responsive to agency (*viz*. treats the model as an entity different from the inanimate substrate, producing an outcome that can be emulated); (ii) ‘mirrors’ the acts of the model with some degree of fidelity, as in so-called ‘true’ imitation (see [3]); (iii) follows the displacement of components of the scene regardless of any sensitivity to demonstrator agency, as in object movement re-enactment [4]; and/or (iv) is affected by local and/or stimulus enhancement. The fidelity of copying, for instance, can be revealing of how the actions or the goals of others are encoded and drive the behaviour of observers.

In mammals, studies of the social transmission of tool use are rare (e.g. [5]) except in the case of primates (principally chimpanzees), where there is extensive experimental and observational evidence that events of tool-use acquisition can be transmitted to conspecifics, both in captivity and in the wild (e.g. [6–16]). In birds, social transmission of tool use has been mainly addressed in two species where tool use is an inherited adaptation, namely woodpecker finches and New Caledonian crows (NCC) [17–20]. Social learning does not seem to play a critical role in the developmental onset of tool use in either of these species: juveniles exhibit tool use independently of the presence of a demonstrator during their ontogeny [17,18]. Although NCCs develop tool use without models, social learning seems to enhance their tool-oriented behaviour: captive-reared subjects allowed to observe tool-use demonstrations by human carers show higher rates of handling and inserting sticks into holes than naive counterparts [19]. Also, wild populations of NCCs show geographical variation in tool morphology [20] and a demographic structure consistent with the existence of a physical culture [21]. This limited information implies that, at least in the case of species with a heritable disposition to tool use, social transmission may not be responsible for the emergence of the behaviour, but may influence its development and topography.

The social transmission of physical skills, including tool-related behaviour, is even less well understood in species that do not normally use tools in the wild. There is some evidence that captive kea pay attention to social cues in a problem involving the insertion of a ball into the correct apparatus in a two-choice task, but the details of what was transmitted are uncertain because this was only studied after the animals had already accomplished the insertion of balls into tubes for food [22].

Recently, a new case of innovative avian tool use and manufacture was reported in a captive male Goffin cockatoo named Figaro. This individual spontaneously discovered how to make and use elongated splinters cut out of a wooden beam. He sculpted the splinters as necessary by adjusting their dimensions in order to retrieve play or food objects out of his reach [23]. This can be treated as an individual innovation because Goffin cockatoos are not known to make or use tools, which for them is ergonomically difficult. The curved shape of their beaks impedes their holding of objects facing forward, whereas their dexterity in using their claws, beak and tongue means that they can solve most physical problems involving physical manipulations using different parts of their own body rather than tools [24].

Figaro's case offers the opportunity to explore whether individually acquired tool competences can be socially transmitted in a non-habitually tool-using bird, and, if this is the case, by what mechanism.

Here, we use the subject that had previously shown spontaneous tool manufacture as a model in a social transmission study to explore whether observing his behaviour triggers tool use in other conspecifics and to investigate what type of observation is responsible for others acquiring tool behaviour, aiming at identifying which social learning mechanisms need to be invoked. To explore sensitivity to agency and the relative roles of imitation, emulation and stimulus enhancement, we compare the effect of observing (i) full demonstrations, (ii) the action of ‘ghost’-operated tools (i.e. tools moving apparently on their own) and (iii) ghost-delivered food (i.e. observing the same conspecific receiving and eating food rewards without any action). Furthermore, we explore the generalization of a socially acquired tool-use skill to the problem of tool manufacture.

## 2. Material and methods

### (a) Subjects

Twelve Goffin cockatoos, six males and six females, participated as observers (electronic supplementary material, §1) and another, Figaro, served as a demonstrator. All subjects had been hand-raised and kept in a large, enriched group aviary (indoors: 45 m^2^ ground space, 3–6 m high wall to gable; outdoors: 150 m^2^ ground space, 3–4.5 m high); a selection of fresh foods, mineral sources and drinking water were available ad libitum. The indoors area was kept above 20°C from October to May. All birds were individually marked with coloured leg bands. Subjects had not been experimentally exposed to Figaro's (or any other) tool use (except for a female, Heidi, that approx. 12 months earlier had witnessed Figaro's tool use and was at the time given a single opportunity in the original setting; at that time she failed to retrieve food) [23]. As they were housed communally, we cannot exclude the possibility of Figaro having displayed some playful tool use within the group. This uncertainty means that there is a possibility that participants were not totally naive, but does not compromise potential differences between the groups. Data were collected from March to July 2013.

In the wild, Goffins inhabit the small Tanimbar archipelago (5082 km^2^) in Indonesia. As with all species of the genus *Corella* [25], the Goffin is likely to be a feeding generalist. Observations during population counts indicate that their social organization may include bonded pairs, family groups including juveniles guided by their parents and nomadic sub-adult flocks of between 10 and 100 subjects [26]. Within pairs, the male feeds the female during incubation [27].

### (b) Apparatus and procedure (social transmission of tool use)

**Figure 1.**
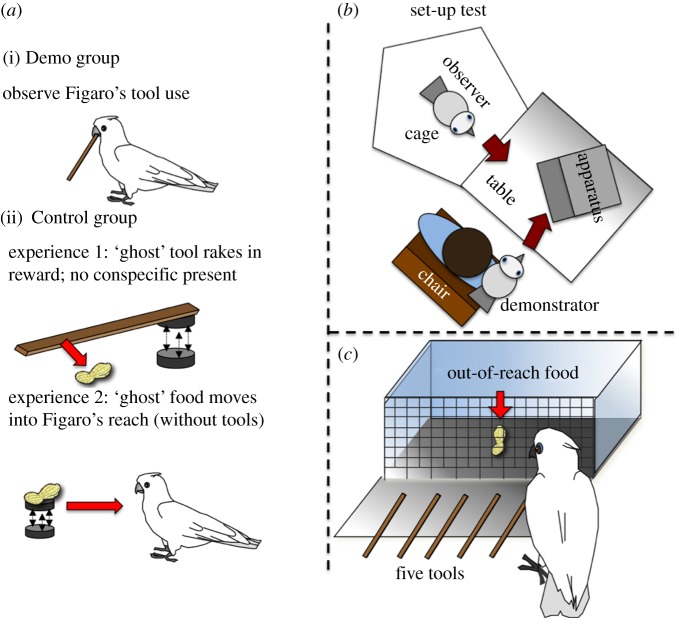
(*a*) The three kinds of demonstration. (i) Demo group, with Figaro using the tool to rake in and eat the food; (ii) Control group, experiencing first a magnetically controlled ‘ghost’ tool raking in the food out of the apparatus, and later the magnetically controlled ‘ghost’ food item moving towards Figaro and being consumed by him. (*b*) Plant view of the set-up. The observer is inside a cage, facing the obliquely placed food-containing apparatus. During demonstrations, a tool-using cockatoo (Figaro) is released on the table, where he reliably uses a tool to extract the food. (*c*) A subject being tested. Food is inside the apparatus, out of the subject's reach, and five potential tools (13 cm) are available.

The main apparatus was a transparent acrylic box (25 cm wide, 20 cm deep, 15 cm high) with a wire grid front (19 × 19 mm; 0.2 cm strong). In the bottom row of the grid every second vertical wire was cut out (figure 1). The box stood on an aluminium floor and was placed on a table (1 × 1 m). Before each trial, a food reward (1/4 of a cashew nut) was placed at the centre of the box, out of the subjects' direct reach. Five wooden strips (approx. 13 cm long, approx. 3 mm wide, 0.5 mm thick) were placed alongside the apparatus during testing and, in the Demo group, during demonstrations (see below).

All 12 subjects received first an ‘in-private baseline trial’ of 15 min with the baited apparatus and the wooden strips. As described in the Results section, none of the birds used the tools successfully at this stage. After this, six subjects (three males and three females) were randomly assigned to one of two groups, labelled ‘Control’ and ‘Demo’. In both groups, every testing session was preceded by three exposures that differed between the groups. If and when a subject used a tool successfully, this procedure changed as explained later. During exposures, the subject was placed in a parrot cage in front of the experimental table (figure 1). In the Demo group, the exposures were demonstrations by Figaro using one of the tools provided to rake in and eat the food placed in the box (figure 1). The Control group experienced two different kinds of exposure, first, the ‘magnetic tool experience’ (MTE) and then the ‘magnetic food experience’ (MFE). During MTEs, a ‘ghost’-operated tool, moved by a magnet under the table, raked the food out of the apparatus, without Figaro being present. During MFEs, no tools were present, instead subjects observed how the food reward (placed on a small metal dish) moved, driven by a magnet under the table, into Figaro's reach, and thereafter Figaro eating it. Members of the Demo group received five sessions in total. Members of the Control group first received five testing sessions preceded by MTE and after that five testing sessions preceded by MFE. A session lasted up to 15 min or until the food was retrieved (labelled a ‘success’). Upon a first success subjects received up to nine further trials within the same session, with number of trials contingent on the observed events, as explained later. In order to maintain attention, subjects were rewarded with a small piece (approx. 1/8) of a cashew nut each time Figaro started to eat in full demonstrations or in MFEs, or after the food had exited the grid in MTEs.

### (c) Apparatus and procedure (tool manufacture)

Two subjects that had succeeded in retrieving food with the provided tools were tested on a manufacture task. The same basic apparatus was used, but no suitable potential ready-made tools were available. Instead, a block of larch wood (0.5 cm deep, 18.0 cm long, 6.5 cm wide) was present alongside the apparatus. Subjects were first tested in this situation in sessions lasting up to 15 min, stopping upon success.

One subject (Dolittle) started to manufacture tools in his fourth session (see Results), but the other (Kiwi) did not, and after his fifth session was exposed to an additional session preceded by three tool-making demonstrations by Figaro.

If a subject started to make and use tools, it received up to nine more trials within the same session (provided it kept succeeding). Testing ended after two successful sessions totalling 20 consecutive successful trials.

### (d) Analysis

All sessions were videotaped and analysed from the videos. For each social transmission of tool-use session, we recorded the number of times the potential tools were picked up, the durations of contact between tool and the front wire grid, and whether the subject was successful in raking in the nut using the tool. As trial duration varied, all counts were analysed per minute of trial time. In successful sessions (which incorporated more than one trial), we calculated an average across trials. We used a generalized linear mixed model in IBM SPSS to control for the effects of ‘sex’ and ‘treatment’. As it was evident from the raw data that there was no possible significant difference in performance between either of the two experiences of the control group, ‘order’ was not added to the model; see the electronic supplementary material, §4. ‘Subjects’ were entered as a random effect [28]. As the data did not meet the criteria for parametric analysis, non-parametric statistics were used for post hoc testing. In the manufacture task, we recorded the number and size of all pieces of wood (stripped from the larch block) that were combined with the grid, the time taken to manufacture successful tools and to retrieve the food, and which pieces of wood were successfully used to retrieve the reward.

As there were two people scoring the videos in equal parts (S.W. and A.S.), 20% of the data were randomly selected and re-analysed for inter-observer reliability (Pearson's correlation coefficient of more than 96% for all parameters measured). We used non-parametric, two-tailed statistics and the Bonferroni–Holm method to correct for multiple comparisons.

## 3. Results

### (a) Effects of demonstration on tool-oriented behaviour

In the in-private baseline trial preceding the experiment, some of the birds picked and moved the potential tools, and sometimes placed them in contact with the front grid, but none of them aimed the potential tools towards the food, and none retrieved it. There were no significant differences between the Demo group and the Control group in any of the measured factors, namely mean number of potential tools picked (Mann–Whitney *U*-test, *Z* = 0.73; *p* = 0.5), mean number of combinations of tools with the grid (Mann–Whitney *U*-test, *Z* = 1.89, *p* = 0.18) and total time tools were combined with the grid (*Z* = 1.89, *p* = 0.18).

**Figure 2.**
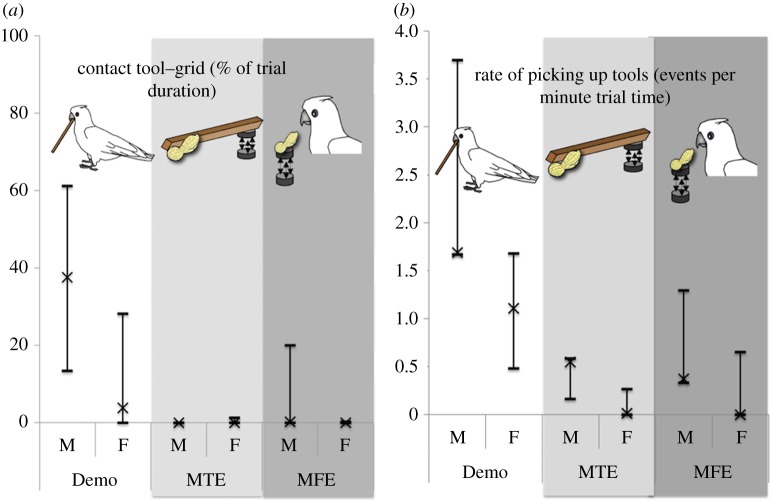
(*a*) Percentage of trial duration when the potential tools were brought into direct contact with the grid. (*b*) Rate of picking up potential tools with the beak or claw (events per minute trial time); for both charts, each line shows the highest, medium and lowest value for each sex (M, male; F, female) within each group (Demo, MTE and MFE).

After the treatments, there was a significant difference between Demo and Control groups for both variables measured (mean pick-up rate: *F*_2_ = 6.932, *p* = 0.01; duration of grid combinations: *F*_2_ = 4.265, *p* = 0.04) but no significant sex effect in either variable (mean pick-up rate: *F*_1_ = 2.909, *p* = 0.114; duration of grid combinations: *F*_1_ = 1.76, *p* = 0.211) and no effect of an interaction of treatment × sex in either variable (mean pick-up rate: *F*_2_ = 0.694, *p* = 0.519; duration of grid combinations: *F*_2_ = 1.604, *p* = 0.241). The mean number of tools picked up was higher in the Demo group than in the Control group, both when the latter were scored after completing the MTE phase (Mann–Whitney *U*-test, *Z* = 2.56, *p* = 0.009) and after the MFE phase (Mann–Whitney *U*-test, *Z* = 2.4, *p* = 0.015). There was no significant difference between the two phases of the Control group in mean number of material pick-ups (paired Wilcoxon signed-rank test, *Z* = 0.94, *p* = 0.44), even though by the time of MFE they had accumulated more experience (figure 2).

The mean duration of combining the tool with the grid was significantly longer in the Demo group than in the Control group after the MTE (Mann–Whitney *U*-test, *Z* = 2.32, *p* = 0.02) but no longer so after the MFE (Mann–Whitney *U*-test, *Z* = 1.77, *p* = 0.091). This is the only hint that the control animals may have been increasing their performance, as there was no difference in the duration of combining tools with the wire grid between MFE and MTE (paired Wilcoxon signed-rank test; *Z* = 0.73; *p* = 0.63). We could not quantify any effect of repeated exposure, as there was no significant change in any of the parameters measured across sessions (Friedman tests, *χ*² < 9.9, *p* > 0.05).

### (b) Social transmission of tool use

All three males in the Demo group acquired the ability to use tools to retrieve food after four or five demonstration sessions: one (Dolittle) succeeded for the first time in session four, failed in the second trial of the same session, but later succeeded in all 10 trials of session five. A second one (Kiwi) started in his fifth session and succeeded in retrieving the food for three consecutive trials, and the third one (Pipin) started in session four and continued in all but one of the remaining trials of sessions four and five. No other subject succeeded in raking in the nut in the course of this experiment.

**Figure 3.**
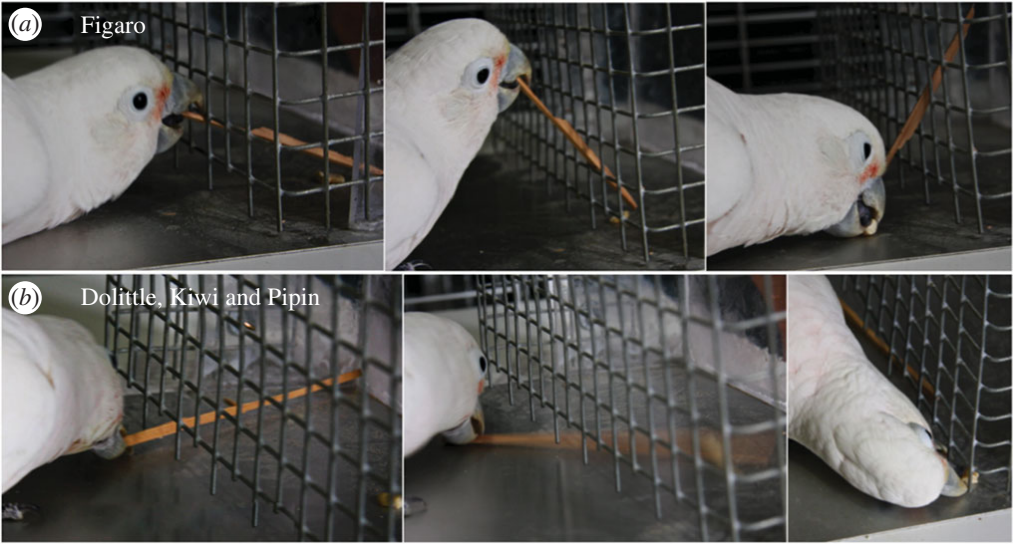
(*a*) Figaro uses the tool as a rake, adjusting the functional end to the changing position of the food reward (*b*) Dolittle, Kiwi and Pipin (left to right, respectively) use the tool as a lever, inserting it along the bottom slot, and swinging the tool laterally, pushing the reward sideways out of the box with a quick flipping movement.

To establish the nature of the social influence, it is crucial to examine the fidelity with which observers replicate the behaviour of the demonstrator. The three successful subjects did not replicate the demonstrator's actions with much fidelity. First, they differed in how they held the tool while inserting it through the grid: while two of them (Dolittle and Kiwi) always held the tool between upper mandible and tongue or between both mandibles as Figaro did, the other one (Pipin) developed an idiosyncratic procedure, placing the tool on the ground and then sliding it gradually forwards through the grid by pushing it with movements of his tongue (electronic supplementary material, §2 and §3, and movie S1). Second, the pattern of combining the tools with the target food differed considerably: while Figaro inserted the tools at different grid heights, constantly adjusting the tool's tip position as the reward moved, the three observers kept the tools on the ground and flipped the food out with a rapid, levering movement caused by applying torque to the proximal end of the tool between the upper and lower mandibles (figure 3; electronic supplementary material, §2 and §3, and movie S1). This flipping movement was facilitated by two features of the testing situation that differed from Figaro's original setting. First, the ground was smooth and slippery, rather than rough wood. Second, there were wider horizontal gaps in the wire mesh at ground level, caused by the removal of vertical wires in alternate cells of the grid.

Although none of the females succeeded and seemed less motivated than the males towards the task, those in the Demo group made some progress (electronic supplementary material, §4), while the Control females did not. One of them kept pushing the stick horizontally on the floor along the grid with enough force to occasionally break it against the wires.

### (c) Tool manufacture

**Figure 4.**
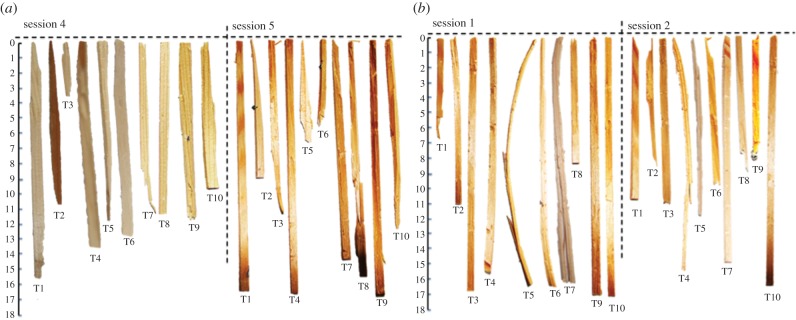
Manufactured tools in trials 1–10 (T1–T10) used to successfully retrieve the food reward in (*a*) Dolittle's and (*b*) Kiwi's two successful sessions. (Online version in colour.)

One of the three observers that succeeded in using the provided tools, Pipin, started to display reproductive behaviour, and could not be separated from his mate for testing any further. Another (Dolittle) spontaneously made and used a tool in the fourth manufacturing session (where tools were not provided but a block of larch wood was available), having seen no manufacturing demonstration. This bird continued making and using the resulting tools for 20 consecutive trials after his first success. The third subject (Kiwi) did not make a tool or retrieved the food within five sessions and was then exposed to manufacturing demonstrations by Figaro (electronic supplementary material, §3 and movie S2). He made a tool and used it efficiently after a single demonstration session and repeated this successfully for 20 consecutive trials after that (see successful tools in figure 4 for both subjects).

Dolittle and Kiwi sometimes lost manufactured splinters after inserting them underneath the grid. In such cases, they often made and inserted fresh ones, making up to seven (electronic supplementary material, §5) before food retrieval in one case. This occasionally resulted in instances of sequential tool use, using a newly made tool to retrieve an earlier one lying out of reach underneath the grid, and using the latter to obtain the reward. This happened in one incident with Dolittle and in seven with Kiwi. In one of these cases, Kiwi used one tool to retrieve a second one, then used the latter to retrieve a third one and finally used this third tool to retrieve the food. In this case, all three tools were of approximately the same length (16.8, 16.9, 17.0 cm, respectively). Both subjects modified the tool once by removing bits that were sticking out, after having had problems inserting it through the grid. In four cases, Dolittle made splinters that were obviously too short given the position of the food, and carried them to the front of the apparatus, but then discarded them without even trying to insert them. In all four cases, he went back to manufacture splinters that were of sufficient length.

Qualitative observations of the manufacturing process employed by Dolittle and Kiwi also showed differences (electronic supplementary material, §6).

## 4. Discussion

Our main finding is that having failed to innovate tool use spontaneously, three male Goffin cockatoos exposed to a male tool-using conspecific acquired and sustained this competence. By contrast, three females that had received the same treatment and six other birds of both sexes that had been exposed to ‘ghost’ demonstrations including similar movements of tools and food, failed to successfully use tools. The three females that observed social demonstrations seemed to advance more with the material provided than the birds in the control group, even if they did not ultimately succeed. To our knowledge, this is the first controlled experimental verification for social acquisition of tool use in a bird species, and one not known for having tool-related adaptations. While NCC appear to refine their tool use through social influences, they all acquire functional tool use at an early age even in the absence of social exposure [18,19,29].

Two of the three males that had learned how to use tools (the third could not participate) thereafter acquired tool-manufacturing skills. One of them did so by simply being exposed to potential tool material, and the other after observing tool-manufacturing demonstrations by a demonstrator.

Our observations address several issues of interest to the comparative cognition of tool use. First, while tool-related innovation by a single member of the species had been previously reported [23], these observations corroborate the cognitive and physical capabilities for tool-related behaviour in this species, in the absence of reinforcement.

Second, they illustrate the complexity of unravelling individual or sex differences in innovative behaviour. We cannot explain as yet why a single male subject (Figaro) invented both manufacturing and use of tools spontaneously, whereas others (Pipin, Dolittle and Kiwi) acquired tool use but only after observing the innovator.

Regarding potential sex differences, we must be cautious because sample sizes preclude robust statistical conclusions, and as the only demonstrator was male the effects of the sex of demonstrator and observers is confounded. However, it is suggestive that all males in the demonstration group but none of the females showed successful acquisition. Such differences may be caused by multiple mechanisms. For instance, differential social attention causes sex differences in some mammals (e.g. [30–32]). Similarly, differences in foraging niche between the sexes (*viz.* male Goffins feed the females during incubation [25,27]) may cause differences in foraging-related cognition. Whatever be the mechanism, the suggestion of sex differences in the present data calls for tests that require training females to use tools in future designs (e.g. [31,32]).

The third and perhaps most important issue refers to the mechanisms for social transmission. There are important distinctions between copying action patterns after observing a model animal, being motivated to achieve the same result as the model, or simply directing attention to the site or stimuli involved in the model's behaviour. Such distinctions are of interest because they are likely to be supported by different cognitive operations. In the present context, the two most likely categories are imitation or emulation. The former places emphasis on matching the demonstrator's actions, the latter on achieving the demonstrator's results [33–36]. In studies where observers choose between two discrete alternatives, imitation involves following the model's choice with an above chance probability (e.g. [37,38]).

We could not apply a clear-cut two-action design as we had only one tool-using demonstrator available. Nevertheless, as our observers only achieved the result after watching demonstrations, and as their techniques differed substantially from that of the model, emulation is a more likely candidate mechanism than imitation: unlike the demonstrator, all three successful observers used a levering rather than a raking technique to obtain the reward. Their technique was arguably easier to operate on the smooth substrate on which they acquired the behaviour than was the demonstrator's raking, which had been acquired on a rough aviary beam and with a shorter tool. Although not likely, we cannot fully dismiss the possibility of low-fidelity imitation of part of the problem, as two of three successful observers used the same insertion (holding the tool at the distal end) technique as the model.

Huang & Charman [39] subdivide emulation into four categories. In *object movement re-enactment*, the observer perceives the object's movement and the corresponding outcome, which in turn stimulates it to reproduce the outcome. In *emulation via affordance learning*, the subjects perceive stimulus consequences, such as ‘dynamic properties and temporal–spatial causal relations of objects, through watching the object movements’. Either type of observational learning would have led to a similar performance between the demonstration group and the control subjects that saw ghost tools raking in the food (MTE). In *end state emulation*, observing the end result prompts the subject to reproduce that state, without reference to the demonstrator's actions to achieve that state. This would have led to absence of differences between the demonstration group and the MFE control, in which the birds saw the demonstrator eating food that had travelled autonomously into its reach. Finally, in *goal emulation*, the observer attributes a goal to the demonstrator but develops its own strategy to replicate the end result. To avoid the obvious difficulties of demonstrating attribution of psychological states, Whiten *et al*. [40] further differentiate between *goal emulation* and *result emulation.* In the latter, the observer reproduces the results of the model's action without necessarily requiring goal attribution*.* This category is compatible with most of our findings: birds in the Demo group may have perceived Figaro as an active agent causing tools to bring food into reach, but may not have paid attention to his exact movements. This may have heightened their motivation and persistence leading to independent discoveries. While it is not possible to resolve many of these issues, result emulation is our strongest candidate mechanism. Testing with demonstrators using various different tool-use techniques would help in addressing this problem further.

Tool use is of course different from tool making. We could explore the latter in only two of the three subjects that had become competent tool users, because the third became unavailable. Both succeeded in making and continuing to use tools, and after the first such success they unfailingly made and used their own tools. One of them acquired tool making spontaneously, and the other after just one demonstration session. During the tool-making part of the study, we observed both sequential tool use and tool modification. Lost tools were retrieved with other tools even if they were no more suitable for the needs of the task than the one in possession of the birds, showing an ability to use tools on non-food items, but leaving uncertain their ability to do this according to functional needs. Observations of tool modification, such as breaking off bits impeding a tool's insertion, or selectivity, as when discarding insufficiently long tools without trying to insert them and then proceeding to make a suitable long tool, indicate some functional apprehension of the affordances of the problems faced.

In conclusion, our findings indicate that tool use can be socially transmitted in Goffin cockatoos, most likely via emulation learning. The competence for using tools may by itself scaffold the discovery of tool manufacture. The existence of qualitative and/or quantitative differences in tool-related cognition between species possessing heritable tool-related behavioural adaptations and those that acquire these competences by individual or social innovation is a tantalizing but unresolved area for comparative cognition research.

## Supplementary Material

Word File containing 1) supplementary information on subjects, 2) supplementary details on individual insertion techniques, 3) Online links to Movie files for Movie S1 & S2, 4) Supplementary individual data for social learning experiment, 5) Images of all manufactured material during manufacture experiment

## Data accessibility

Raw data set available on Datadryad.org (doi:10.5061/dryad.5hk2h).
