# Supplementary material for: Social transmission of tool use and tool manufacture in Goffin cockatoos (Cacatua goffini)
Source: Proc Biol Sci. 2014 Oct 22;281(1793):20140972. doi: 10.1098/rspb.2014.0972 (PMC4173672; doi:10.1098/rspb.2014.0972)
Supplement: Word File containing 1) supplementary information on subjects, 2) supplementary details on individual insertion techniques, 3) Online links to Movie files for Movie S1 & S2, 4) Supplementary individual data for social learning experiment, 5) Images of all manufactured material during manufacture exp [file rspb20140972supp1.docx]

**Electronic Supplementary Material**

1. **Subjects**

Subjects’ treatment, name, sex, and hatching year. All subjects were hand raised.

| ***Group*** | **Subject** | **Sex** | **Hatched** |
| --- | --- | --- | --- |
| *Demonstrator* | Figaro | M | 2007 |
| *Demonstration*  *Group* | Pipin |  | 2008 |
|  | Kiwi | M | 2010 |
|  | Dolittle |  | 2011 |
|  | Fini |  | 2007 |
|  | Heidi | F | 2010 |
|  | Moneypenny |  | 2010 |
| *Control Group* | Konrad |  | 2010 |
|  | Muppet | M | 2010 |
|  | ZoZo |  | 2010 |
|  | Ladybird |  | 2010 |
|  | Olympia | F | 2010 |
|  | Mayday |  | 2011 |

1. **Insertion Techniques**

Three different insertion techniques through the grid of the apparatus used by different subjects (Figaro, Dolittle, Kiwi & Pipin) during successful tool use (A-B).


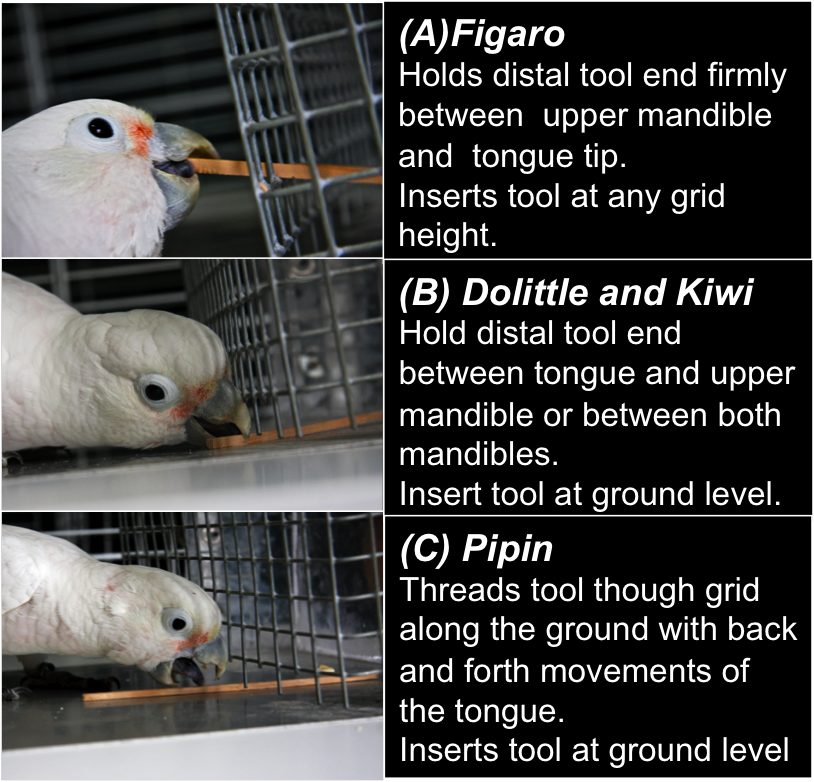


1. **Links to Movies**

Movie S1: Tool use techniques of the demonstrator and the successful observers

http://www.zoo.ox.ac.uk/group/kacelnik/goffin_cockatoos_social_transmission.mov

Movie S2: Tool manufacture example Figaro, Dolittle & Kiwi

http://www.zoo.ox.ac.uk/group/kacelnik/goffin_cockatoos_tool_making.mov

1. **Individual Results Social Learning**

Mean frequency of material pick ups per minute of trial time, and percentage of trial time during which a tool was placed in direct contact with the grid.

| ***Group*** | **Sex** | **Subject** | **frequency**  **pick up**  **material** | **Contact**  **tool-grid**  **% trial duration** | |  |
| --- | --- | --- | --- | --- | --- | --- |
| ***Demo*** | *M* | *Dolittle* | 1.67 | 61.1 | |  |
|  |  | *Kiwi* | 1.69 | 13.3 | |  |
|  |  | *Pipin* | 3.69 | 37.5 | |  |
|  | *F* | *Fini* | 1.11 | 4.0 | |  |
|  |  | *Heidi* | 1.68 | 28.1 | |  |
|  |  | *Moneypenny* | 0.5 | 0.0 | |  |
| ***MTE*** | *M* | *Konrad* | 0.55 | 0.0 | |  |
|  |  | *Muppet* | 0.59 | 0.0 | |  |
|  |  | *ZoZo* | 0.16 | 0.0 | |  |
|  | *F* | *Ladybird* | 0 | 0.0 | |  |
|  |  | *Mayday* | 0.27 | 1.2 | |  |
|  |  | *Olympia* | 0.01 | 0.0 | |  |
| ***MFE*** | *M* | *Konrad* | 1.29 | 20.0 | |  |
|  |  | *Muppet* | 0.37 | 0.0 | |  |
|  |  | *ZoZo* | 0.33 | 0.2 | |  |
|  | *F* | *Ladybird* | 0 | 0.0 | |  |
|  |  | *Mayday* | 0.65 | 0.2 | |  |
|  |  | *Olympia* | 0 | 0.0 | |  |
|  |  |  |  |  |  | |

1. **Manufactured Material**

Manufactured material inserted through the grid front of the apparatus during the tool manufacture phase by subjects Dolittle and Kiwi. Y-axis= material length in cm; T1-10 = Trials 1-10; Material pieces which were successfully used to retrieve the food from the box (Tools) are marked in green. Sessions in which no material pieces were manufactured are omitted.


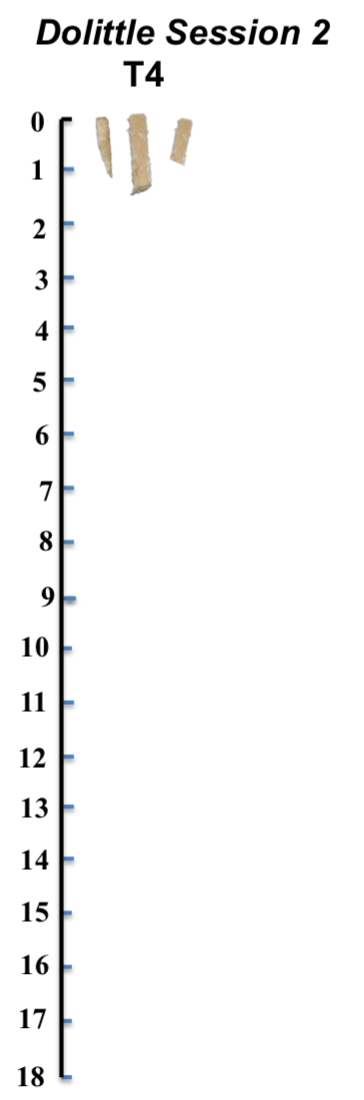


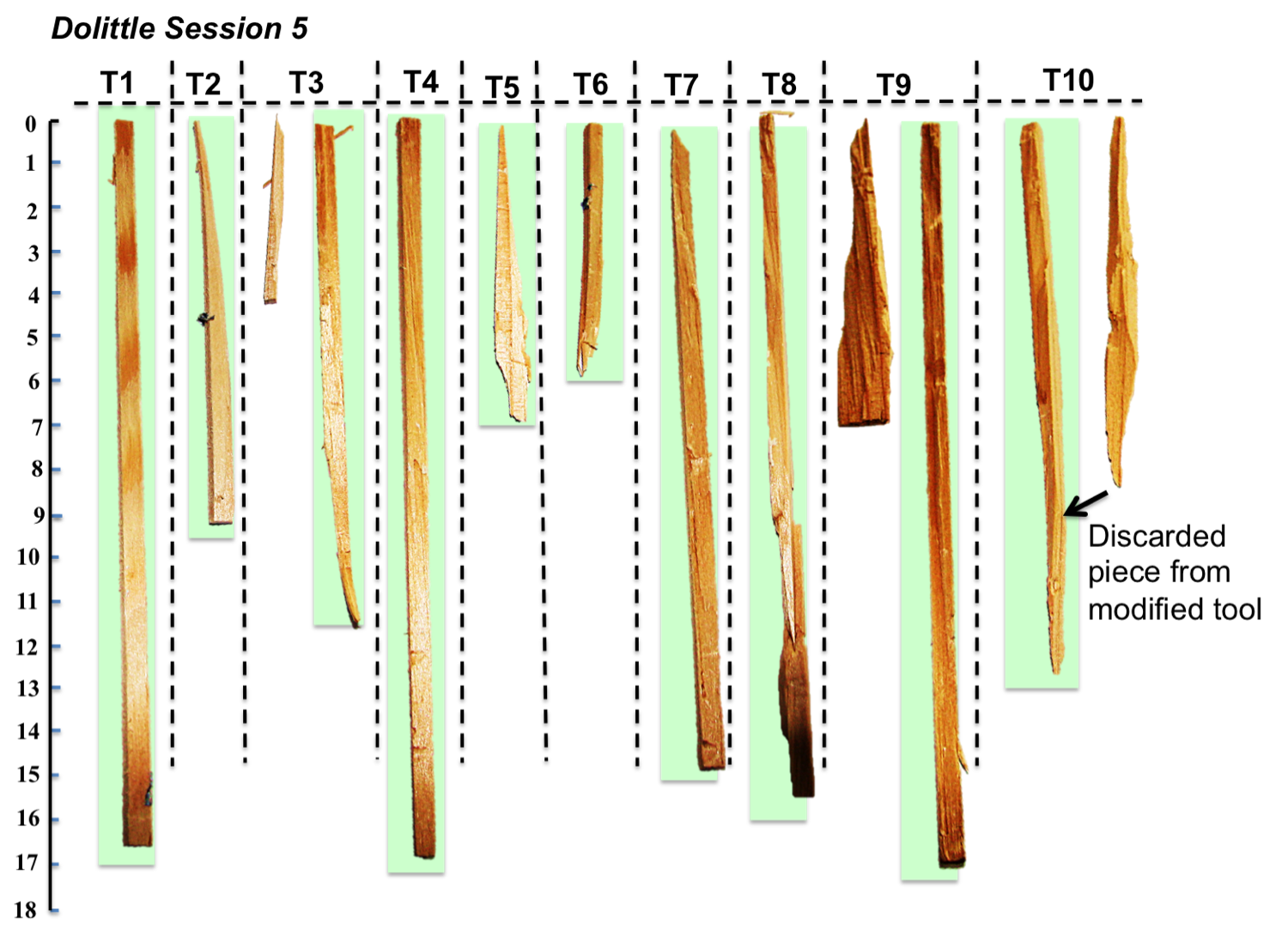


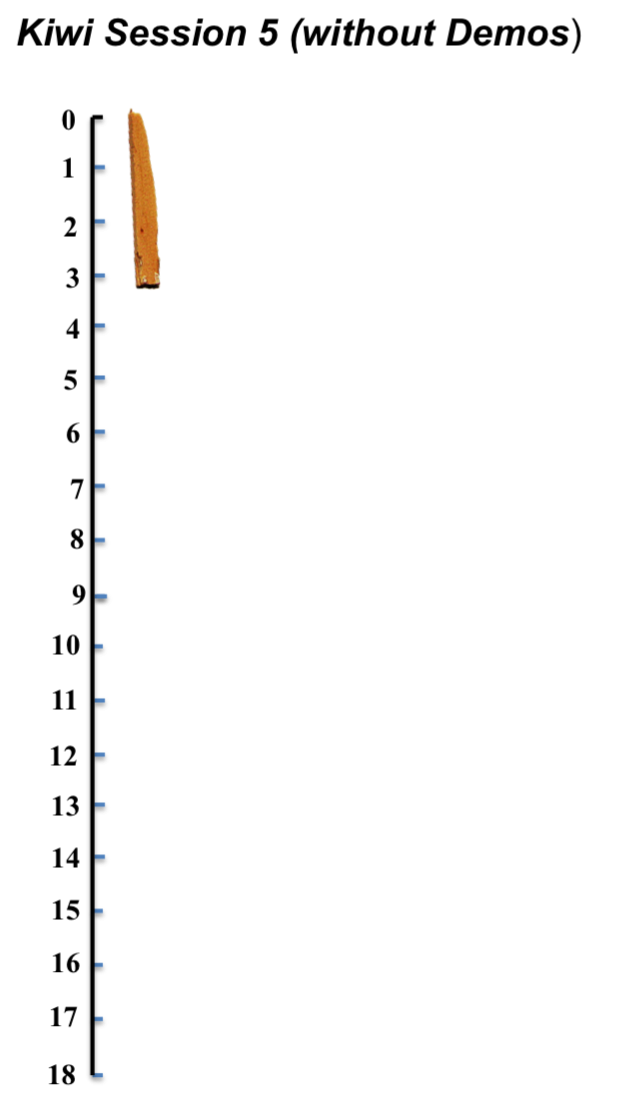


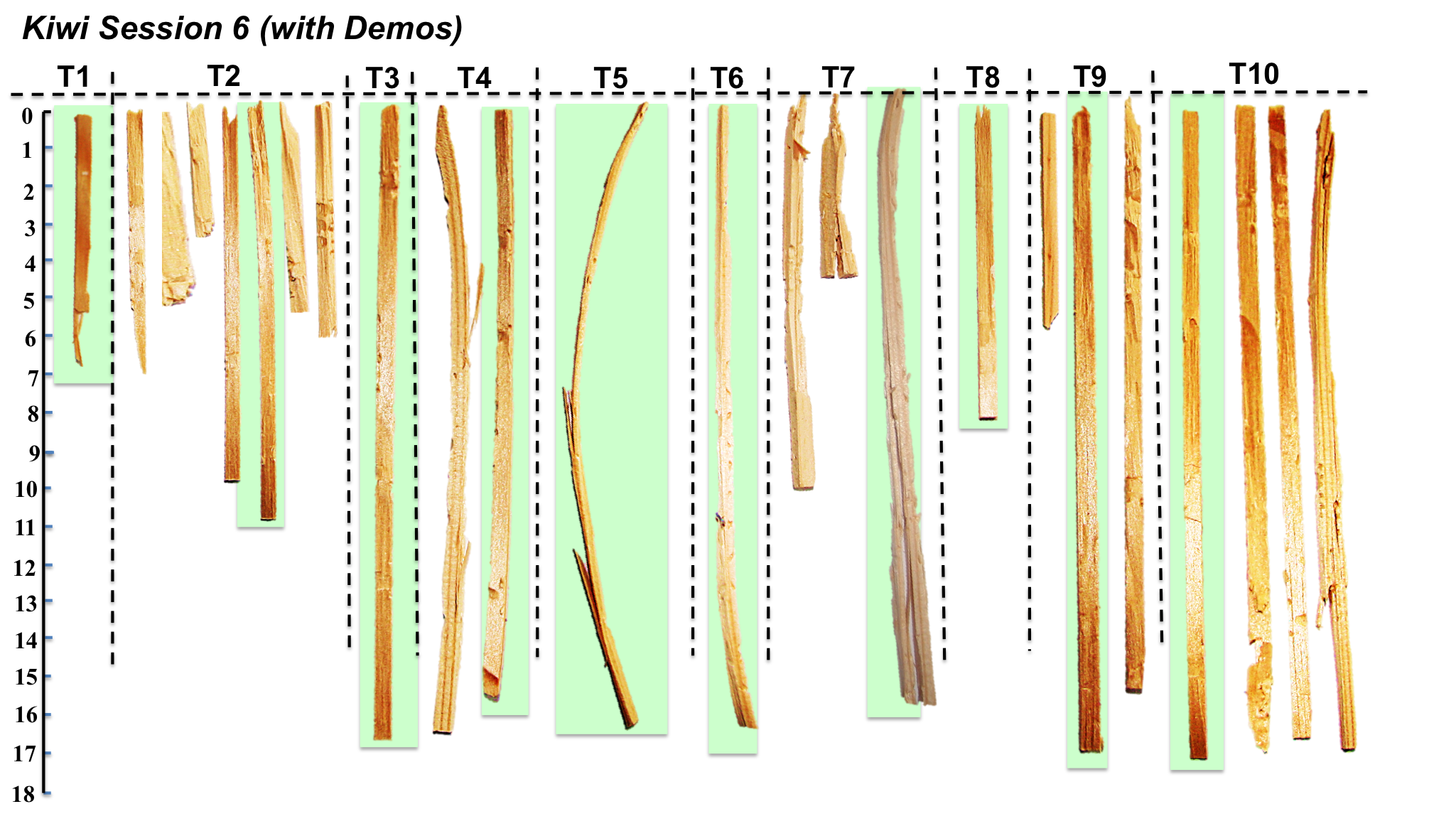


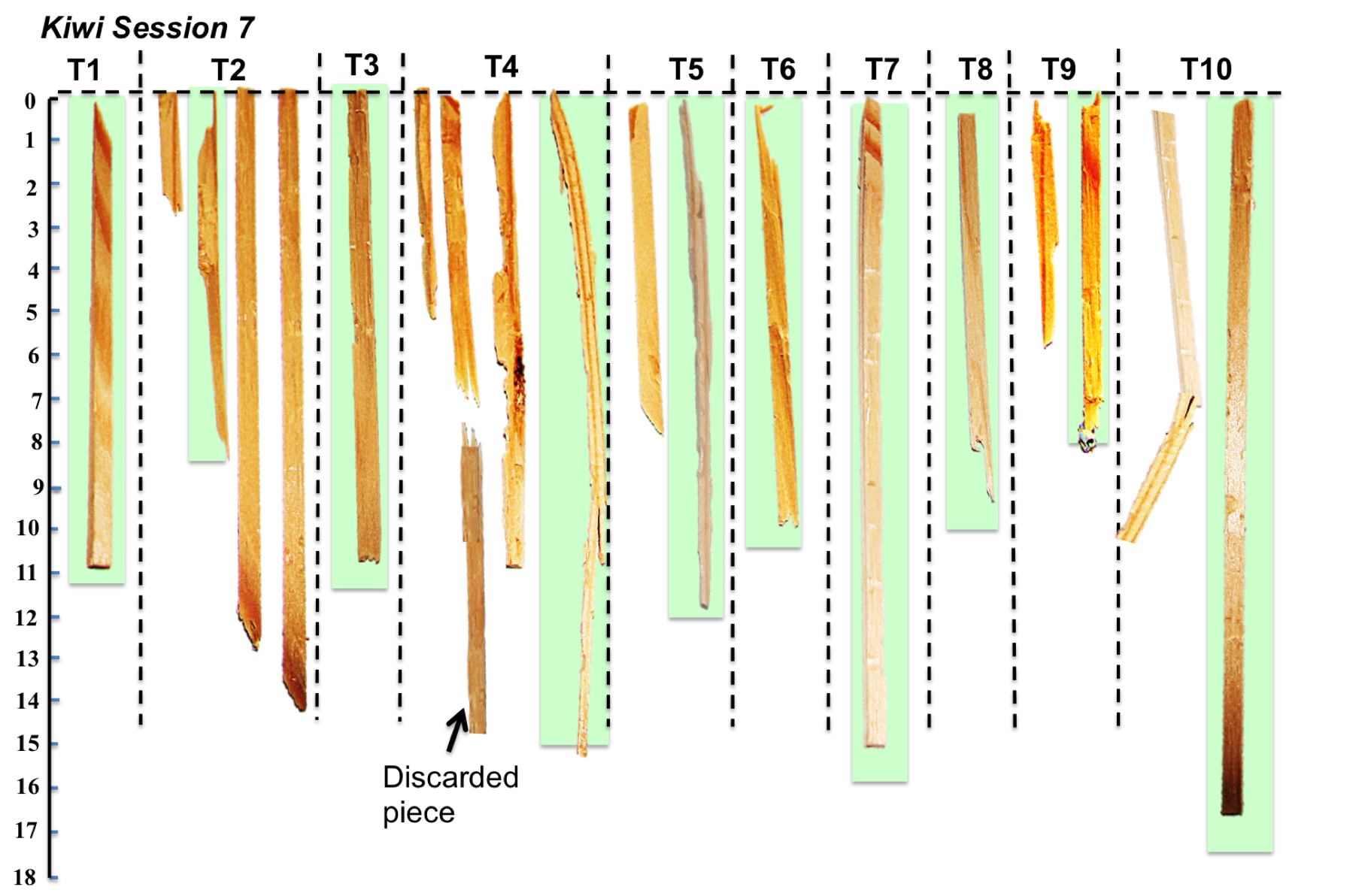


1. **Individual differences in tool manufacture**

There were no significant differences between Dolittle and Kiwi in length of the successful tools (Mann-Whitney U test, Z=0.49, p=0.64), number of material pieces inserted through the grid (Mann-Whitney U test, Z=0.22, p=0.84) or total trial time until success (Mann-Whitney U test, Z=0.758, p=0.45). However, Kiwi made his successful tools in half the time it took Dolittle (Average Kiwi (in sec) = 14.5 +/- 4.47 SE; Dolittle=30.05+/-4.5 SE; Mann-Whitney test, Z=3.45, p<0.0001).

Dolittle inserted fewer pieces of material into the grid before retrieving the reward in his second successful session than in his first (Wilcoxon test, Z=2.12; p=0.034; see ESM), while no such improvement was found for Kiwi (Wilcoxon test, Z=1.063; p=0.289). Kiwi’s successful tools were slightly longer in the first than in the second session (Wilcoxon test, Z=2.73, p=0.035; see Figure 3), no such difference was apparent in Dolittle (Wilcoxon test, Z=0.866, p=0.386; see Figure 3). We found no difference in performance between the first and the second session in time to manufacture the successful tools (Wilcoxon test, Dolittle Z=0.153, p=0.878), Kiwi Z=0.178, p=0.859). Dolittle, however, (unlike Kiwi, Wilcoxon test, Z=0.561, p=0.58) was faster in his second than in his first successful session in the total trial time required to obtain the reward (Wilcoxon test, Z=2.5, p=0.012).
